# Supplementary material for: Characterization of Novel Rhabdoviruses in Chinese Bats
Source: Viruses. 2021 Jan 5;13(1):64. doi: 10.3390/v13010064 (PMC7824899; doi:10.3390/v13010064)
Supplement: Supplementary file 1 [file viruses-13-00064-s001.zip › Figure S1.pdf]

Figure S1. Sequences of the transcription initiation (TI) and the transcription termination (TTP) signals of the 6 Chinese bat rhabdoviruses gene junctions. Conserved motifs are indicated in grey for TI or underlined for TTP. The consensus sequences are indicated, with Y = C/T, M = A/C, W = C/G and R = A/G.

Ledantevirus

Virus	Junction	TTP	Intergenic	TI
TYBV	N-P	CATGAAAAAAA	C	AACGAGG
	P-M	CGTGAAAAAAA	TGTTGCAGATTTTCA	AACGTGG
	M-G	CATGAAAAAAA	C	AACGAGC
	G-L	CATGAAAAAAA	TC	AACGAGG

Vesiculovirus

Virus	Junction	TTP	TI
QZBV	N-P	CATGAAAAAAA	CTAACAGA
	P-M	TATGAAAAAAA	CTAACAGG
	M-G	TATGAAAAAAA	CTAACAGG
	G-L	TATGAAAAAAA	CTAACAGA
	L-trailer	TATGAAAAAAA	
YSBV	Leader-N		TAACAGA
	N-P	TATGAAAAAAA	CTAACAGA
	P-M	CATGAAAAAAA	CTAACAGG
	M-G	TCTGAAAAAAA	CTAGCAGA
	G-L	TATGAAAAAAA	CTAACAGA
	L-trailer	CATGAAAAAAA	
