# Supplementary material for: Characterization of Novel Rhabdoviruses in Chinese Bats
Source: Viruses. 2021 Jan 5;13(1):64. doi: 10.3390/v13010064 (PMC7824899; doi:10.3390/v13010064)
Supplement: Supplementary file 1 [file viruses-13-00064-s001.zip › Figure S2.pdf]

Figure S2

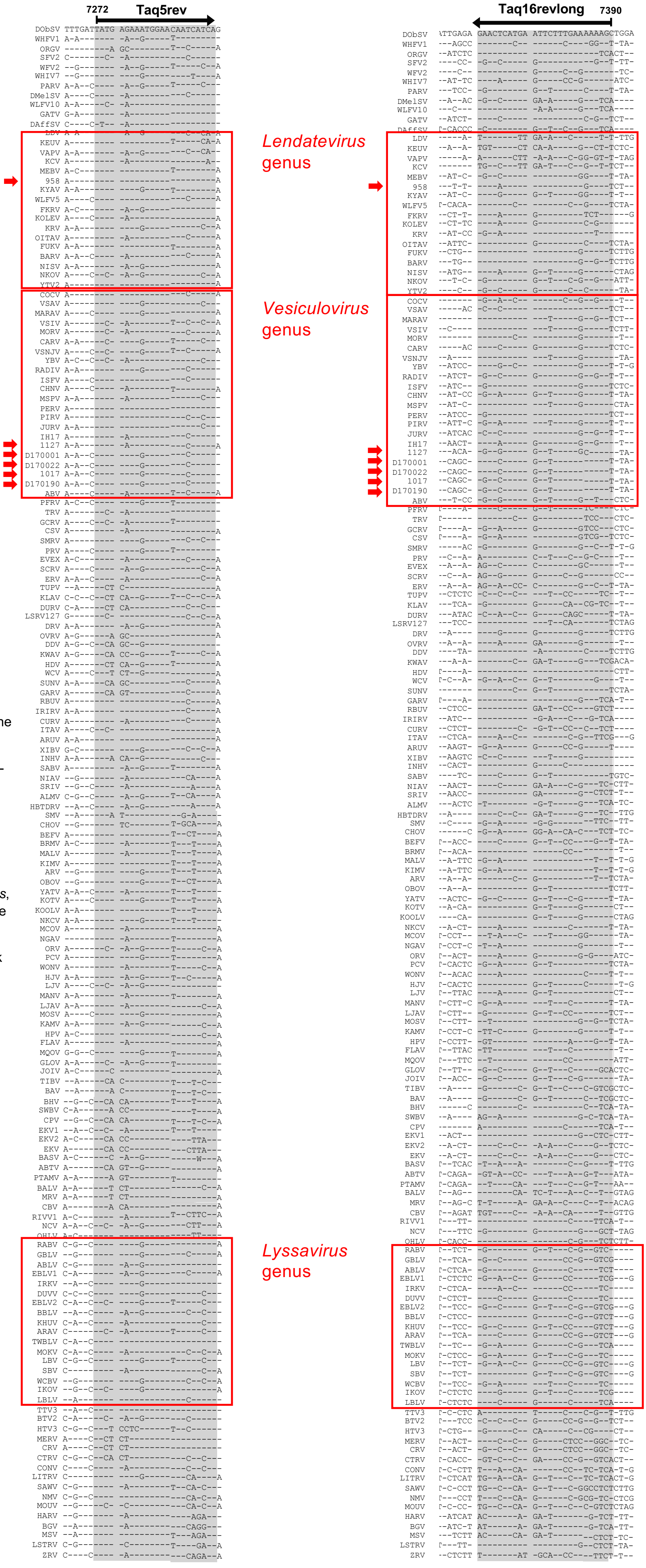

Lendatevirus genus

Vesiculovirus genus

Lyssavirus genus

Legend:
Alignement of a dataset of 161 nucleotide sequences of rhabdoviruses, corresponding to the regions (in grey) targeted by the primers Taq5long and Taq16revlong of the pan-lyssa RT-qPCR [50]. This dataset of sequences is the same used for Figure 3, and the list of the rhabdoviruses is detailed in Table S5. The 6 Chinese bat rhabdoviruses are indicated by a red arrow, and the viruses belonging to the genera *Lyssavirus*, *Vesiculovirus* and *Ledantevirus* are grouped in red square. Positions are indicated according to the reference sequence PV (GenBank accession number M13215).
