# Supplementary material for: Characterization of Novel Rhabdoviruses in Chinese Bats
Source: Viruses. 2021 Jan 5;13(1):64. doi: 10.3390/v13010064 (PMC7824899; doi:10.3390/v13010064)

Figure S3. Validation of the pan-lyssa RT-qPCR used for the screening of bat rhabdovirus, with isolate D170001 (YSBV virus) as an example. The limit of detection with the standard curve used (serial dilutions of plasmids in nuclease free water) is indicated in red (number of target copies per assay), as well as the characteristics of the pan-lyssa RT-qPCR (with indication of R^2 and efficiency values).

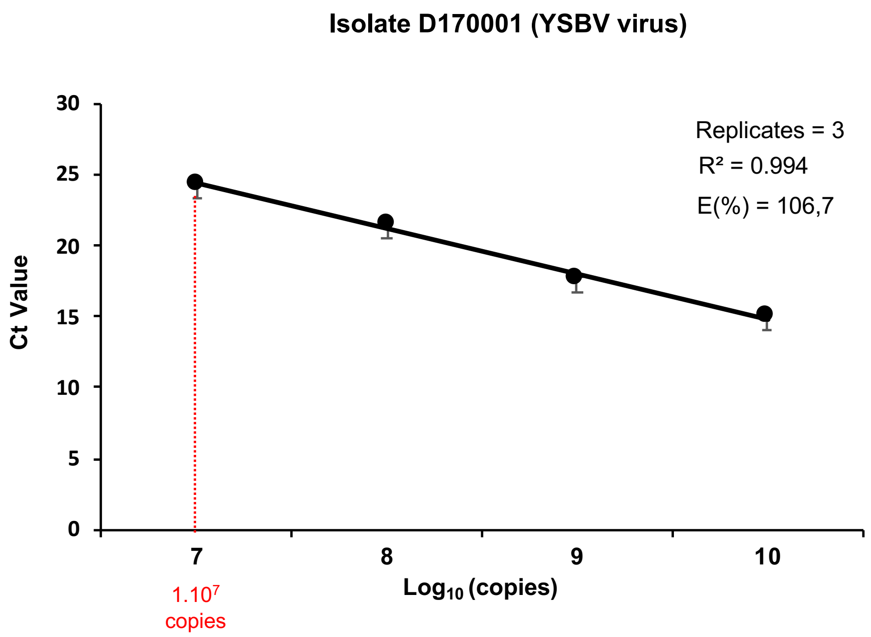

Supplement: Supplementary file 1 [file viruses-13-00064-s001.zip › Figure S3.pdf]
