# Supplementary material for: Characterization of Novel Rhabdoviruses in Chinese Bats
Source: Viruses. 2021 Jan 5;13(1):64. doi: 10.3390/v13010064 (PMC7824899; doi:10.3390/v13010064)
Supplement: Supplementary file 1 [file viruses-13-00064-s001.zip › Table S7.pdf]

Table S7. Amino acid identities of each canonical proteins (N, P, M, G and L) of isolates, 1017, D170001, D170022 and D170190 (putative species YSBV) and isolate 1127 (putative species TYBV) within the *Vesiculovirus* genus. Identities were calculated as pairwise deletion using MEGA7.0. Rhabdovirus isolates described in this study are underlined.

Protein	Pairwise amino acid identity (%)																					
	1127	1017	D170001	D170022	D170190	IH17	ABV	YBV	RADIV	PERV	CHNV	PIRV	ISFV	JURV	MSPV	VSNJV	CARV	VSAV	COCV	MARAV	VSIV	MORV
**N protein**																						
1127																						
1017	90																					
D170001	90	100																				
D170022	90	100	100																			
D170190	90	100	100	100																		
IH17	100	90	90	90	90																	
ABV	55	55	55	55	55	55																
YBV	48	48	48	48	48	48	49															
RADIV	44	47	47	47	47	45	49	75														
PERV	49	51	51	51	51	49	54	56	59													
CHNV	43	44	44	44	44	43	48	50	52	59												
PIRV	45	47	47	47	47	45	49	52	54	68	56											
ISFV	45	47	47	47	47	45	49	54	55	63	59	62										
JURV	47	48	48	48	48	47	50	54	57	67	62	63	67									
MSPV	47	48	48	48	48	47	48	53	56	70	59	62	66	71								
VSNJV	47	48	48	48	48	47	47	54	56	62	51	52	53	55	56							
CARV	46	46	46	46	46	46	47	54	55	60	51	50	51	53	56	73						
VSAV	47	47	47	47	47	46	45	54	56	60	49	52	52	55	55	69	75					
COCV	47	47	47	47	47	47	46	53	54	60	49	52	51	53	56	70	74	85				
MARAV	46	46	46	46	46	46	46	53	56	61	51	52	51	54	56	70	73	82	87			
VSIV	48	48	48	48	48	48	46	54	57	60	51	53	52	55	56	69	76	85	84	90		
MORV	47	48	48	48	48	47	47	53	55	60	51	53	52	55	55	69	75	83	83	89	91	
**P protein**																						
1127																						
1017	49																					
D170001	49	100																				
D170022	49	100	100																			
D170190	49	98	98	98																		
IH17	87	52	52	52	51																	

ABV	11	9	9	9	9	11															
YBV	16	15	15	15	14	16	17														
RADIV	13	11	11	11	11	13	16	41													
PERV	14	15	15	15	14	13	20	16	17												
CHNV	14	13	13	13	12	14	18	18	18	29											
PIRV	15	17	17	17	16	14	18	17	18	31	29										
ISFV	15	17	17	17	17	15	17	18	14	31	27	29									
JURV	12	13	13	13	12	13	20	18	19	35	26	34	32								
MSPV	14	13	13	13	13	15	20	14	18	28	28	24	21	24							
VSNJV	15	14	14	14	13	15	17	11	16	23	16	22	18	22	16						
CARV	14	14	14	14	13	14	18	14	19	19	20	27	19	20	20	40					
VSAV	15	14	14	14	14	17	18	18	17	23	22	18	20	15	21	35	47				
COCV	16	15	15	15	15	17	22	19	18	22	22	19	18	19	22	36	47	61			
MARAV	16	15	15	15	15	18	19	19	18	20	19	22	17	16	20	36	49	60	63		
VSIV	15	16	16	16	16	17	19	20	18	20	21	21	19	18	20	33	48	55	59	63	
MORV	17	14	14	14	14	18	20	18	19	20	19	21	17	17	20	34	48	56	63	66	69
**M protein**																					
1127																					
1017	74																				
D170001	74	100																			
D170022	74	100	100																		
D170190	74	100	100	100																	
IH17	95	75	75	75	75																
ABV	41	43	43	43	43	42															
YBV	24	25	25	25	25	24	28														
RADIV	23	25	25	25	25	23	30	56													
PERV	22	24	24	24	24	22	26	29	29												
CHNV	24	25	25	25	25	23	31	32	33	42											
PIRV	25	30	30	30	30	25	34	34	31	51	44										
ISFV	25	26	26	26	26	26	31	35	35	51	51	52									
JURV	24	27	27	27	27	24	30	33	31	50	49	50	54								
MSPV	26	28	28	28	28	27	28	30	33	43	38	44	45	42							
VSNJV	22	20	20	20	20	23	22	25	25	33	28	29	31	33	28						
CARV	22	24	24	24	24	22	23	21	26	29	27	26	27	31	28	55					
VSAV	23	22	22	22	22	23	26	25	26	32	30	31	31	30	31	61	56				
COCV	24	23	23	23	23	24	24	24	25	30	29	31	32	29	29	61	58	81			
MARAV	24	22	22	22	22	24	23	25	27	31	32	31	32	32	29	64	58	77	80		
VSIV	23	23	23	23	23	24	22	25	25	29	29	31	31	30	31	63	56	75	75	80	
MORV	23	23	23	23	23	23	23	24	26	30	32	31	30	32	30	63	61	77	79	83	83
**G protein**																					

1127																					
1017	73																				
D170001	73	99																			
D170022	73	99	100																		
D170190	73	100	99	99																	
IH17	85	73	73	73	73																
ABV	33	31	32	32	31	34															
YBV	27	28	28	28	29	29	26														
RADIV	27	28	28	28	29	28	27	68													
PERV	28	29	29	29	29	28	27	46	44												
CHNV	27	28	28	28	28	27	26	45	44	52											
PIRV	27	29	29	29	29	28	28	48	46	58	52										
ISFV	28	29	29	29	29	28	26	43	42	51	54	50									
JURV	28	29	29	29	29	28	27	45	43	52	53	50	55								
MSPV	27	29	29	29	29	27	28	43	41	49	49	49	49	49							
VSNJV	29	29	28	29	29	29	25	35	35	40	39	39	39	38	38						
CARV	30	30	29	29	30	30	28	38	38	40	42	41	39	39	39	52					
VSAV	30	29	29	29	29	30	23	37	37	41	41	39	38	38	39	49	55				
COCV	31	29	29	29	29	30	24	36	37	39	41	39	42	40	38	48	55	67			
MARAV	31	29	29	29	29	30	24	37	39	40	42	40	40	38	38	51	56	64	74		
VSIV	30	30	30	30	30	30	24	38	38	39	40	39	41	38	37	50	55	63	71	77	
MORV	29	29	29	29	29	29	25	39	39	40	42	39	42	39	39	50	57	64	72	78	85

L protein

1127																	
1017	76																
D170001	76	100															
D170022	76	100	100														
D170190	76	100	100	100													
IH17	91	76	76	76	76												
ABV	58	58	58	58	58	57											
YBV	53	54	54	54	54	53	55										
RADIV	54	55	55	55	55	54	56	72									
PERV	54	54	54	54	54	54	56	59	60								
CHNV	55	55	55	55	55	54	56	60	62	67							
PIRV	54	53	53	53	53	54	58	60	61	67	67						
ISFV	54	55	55	55	55	54	56	59	61	67	69	67					
JURV	54	54	54	54	54	54	58	59	61	65	67	67	67				
MSPV	56	55	55	55	55	55	57	60	61	67	67	68	69	68			
VSNJV	53	53	53	53	53	53	54	57	58	57	58	58	58	58	58		
CARV	53	53	54	54	53	53	55	58	58	59	59	59	59	59	60	70	
VSAV	53	54	54	54	54	53	54	58	58	58	59	58	59	58	59	67	70

COCV	53	54	54	54	54	53	54	58	58	58	58	58	59	58	59	66	69	78			
MARAV	52	53	53	53	53	52	53	58	57	58	59	57	59	58	59	66	69	77	79		
VSIV	53	52	52	52	52	52	54	57	57	58	59	58	59	59	59	66	70	76	77	78	
MORV	54	53	53	53	53	53	54	58	57	59	59	58	59	58	59	66	69	75	77	79	80
