# Supplementary material for: Characterization of Novel Rhabdoviruses in Chinese Bats
Source: Viruses. 2021 Jan 5;13(1):64. doi: 10.3390/v13010064 (PMC7824899; doi:10.3390/v13010064)
Supplement: Supplementary file 1 [file viruses-13-00064-s001.zip › Table S8.pdf]

Table S8. Amino acid identities of each canonical proteins (N, P, M, G and L) of isolate 958 (putative species QZBV) within the *Ledantevirus* genus. Identities were calculated as pairwise deletion using MEGA7.0. Rhabdovirus isolate described in this study is underlined.

**Protein**	**Pairwise amino acid identity (%)**																
	958	MEBV	KYAV	WLFV5	OITAV	KRV	KOLEV	FKRV	KCV	VAPV	LDV	KEUV	YTV2	NKOV	NISV	BARV	FUKV
**N protein**																	
<u>958</u>																	
MEBV	87																
KYAV	77	75															
WLFV5	73	73	67														
OITAV	55	55	52	52													
KRV	61	60	58	58	58												
KOLEV	61	62	60	62	58	63											
FKRV	60	60	58	57	56	60	74										
KCV	37	38	36	37	39	38	39	37									
VAPV	38	37	37	38	36	35	37	35	48								
LDV	40	41	39	39	39	40	40	37	48	64							
KEUV	42	41	41	40	39	40	39	37	47	63	81						
YTV2	38	40	38	36	36	39	40	40	40	39	39	39					
NKOV	40	40	39	39	38	42	40	40	40	44	40	41	53				
NISV	40	41	39	38	37	40	40	40	39	42	39	40	53	76			
BARV	41	41	39	38	38	42	40	40	40	43	40	40	53	76	94		
FUKV	40	41	40	38	37	41	40	40	40	42	40	40	53	77	95	96	
**P gene**																	
<u>958</u>																	
MEBV	74																
KYAV	27	28															
WLFV5	45	46	29														
OITAV	21	21	20	21													
KRV	17	21	18	23	19												

KOLEV	19	22	20	23	23	20										
FKRV	18	17	20	22	23	18	52									
KCV	16	18	18	15	16	17	16	15								
VAPV	15	17	18	16	16	18	19	17	33							
LDV	14	18	17	18	13	18	19	17	29	35						
KEUV	14	15	18	17	14	18	17	15	30	38	58					
YTV2	16	16	15	17	17	17	14	15	24	23	22	22				
NKOV	15	17	18	16	16	16	18	14	19	23	19	20	23			
NISV	17	19	21	19	18	20	18	15	19	20	19	21	26	40		
BARV	16	17	18	17	16	17	18	15	18	19	19	21	26	42	73	
FUKV	17	19	20	17	16	18	18	16	20	20	19	21	24	42	73	81
**M protein**																
958																
MEBV	66															
KYAV	46	48														
WLFV5	53	49	44													
OITAV	36	36	35	38												
KRV	36	36	32	37	47											
KOLEV	38	36	34	39	54	48										
FKRV	40	38	38	39	54	46	66									
KCV	32	30	27	30	29	29	32	29								
VAPV	31	30	29	30	25	22	28	26	46							
LDV	28	29	25	31	28	25	29	25	44	57						
KEUV	25	28	24	28	25	27	27	27	42	52	64					
YTV2	37	38	34	33	32	34	30	30	32	31	31	30				
NKOV	39	36	36	33	35	34	36	34	30	29	25	28	50			
NISV	39	41	37	36	33	32	35	33	32	33	29	31	57	67		
BARV	39	40	35	36	33	32	34	33	30	32	26	29	57	70	87	
FUKV	40	42	34	36	33	32	34	33	30	32	27	29	56	69	87	96
**G protein**																
958																
MEBV	64															

KYAV	47	47														
WLFV5	45	44	43													
OITAV	31	29	30	30												
KRV	26	26	28	25	33											
KOLEV	31	30	31	31	39	33										
FKRV	32	32	29	29	43	32	51									
KCV	30	31	30	31	25	26	27	25								
VAPV	33	32	31	30	26	25	27	27	51							
LDV	30	31	29	29	24	25	27	27	49	58						
KEUV	31	30	30	30	26	25	26	26	49	57	70					
YTV2	29	31	29	29	27	25	27	27	37	36	33	34				
NKOV	30	28	29	31	26	23	27	26	32	34	31	32	40			
NISV	30	28	29	29	25	24	26	24	33	33	31	32	39	49		
BARV	29	27	29	28	25	24	26	25	33	34	31	31	39	47	78	
FUKV	31	29	29	29	24	25	26	24	34	33	32	32	41	47	78	85
**L protein**																
958																
MEBV	76															
KYAV	63	64														
WLFV5	61	62	59													
OITAV	55	55	53	55												
KRV	55	56	55	55	63											
KOLEV	55	55	54	54	62	63										
FKRV	53	55	53	54	62	62	75									
KCV	48	49	48	48	49	49	49	48								
VAPV	50	50	49	50	50	50	50	49	63							
LDV	48	49	48	49	48	49	48	47	63	69						
KEUV	49	49	48	49	48	49	49	48	63	70	81					
YTV2	48	47	49	47	47	48	48	47	50	49	50	50				
NKOV	50	49	48	48	48	47	48	47	51	51	51	51	55			
NISV	49	49	49	48	48	47	48	48	51	51	50	51	54	61		
BARV	49	49	49	48	48	48	47	48	50	51	50	51	54	61	88	
FUKV	49	49	49	48	48	48	48	48	51	51	50	51	54	62	89	92
